# Asymmetric Sensitivity of Hepatic Venous Pressure Gradient to Portal and Sinusoidal Resistance: An In Vitro Experimental Study

**DOI:** 10.3390/bios16080432

**Published:** 2026-08-08

**Authors:** Jiyang Zhang, Lingjun Liu, Xin Yu, Xiao Li, Zhongyou Li, Taoping Bai, Wentao Jiang

**Affiliations:** 1 Sichuan Province Biomechanical Engineering Laboratory, Sichuan University, Chengdu 610065, China; 2 Department of Mechanical Science and Engineering, Sichuan University, Chengdu 610065, China; 3 Key Laboratory of Birth Defects and Related Diseases of Women and Children, Sichuan University, Ministry of Education, Chengdu 610041, China; 4 Department of Radiology, West China Second University Hospital, Sichuan University, Chengdu 610041, China; 5 Department of Interventional Radiology, Guizhou Provincial People’s Hospital, Guiyang 550002, China; 6 Department of Biomedical Engineering, Faculty of Engineering, The Hong Kong Polytechnic University, Hong Kong, China; 7 College of Computer Science, Sichuan Normal University, Chengdu 610101, China

**Keywords:** hepatic venous pressure gradient (HVPG), portal hypertension, mock circulatory loop (MCL), sinusoidal resistance, global sensitivity analysis

## Abstract

Hepatic venous pressure gradient (HVPG) is the clinical gold standard for assessing portal hypertension, but its dependence on different vascular resistance sources remains unclear. This study evaluated the respective effects of sinusoidal resistance (SR) and portal venous resistance (PVR) on HVPG using an in vitro hemodynamic platform. A mock circulatory loop with dual hepatic blood supply was constructed and calibrated to near-physiological conditions. SR and PVR were independently adjusted to reproduce different portal hypertension states, and HVPG was measured by balloon wedging. A perturbation index (PI) was introduced to quantify the systemic effect of wedging, and Sobol global sensitivity analysis was used to compare resistance contributions. HVPG increased markedly with SR, ranging from 3.69 to 12.25 mmHg, but showed only limited changes with PVR, ranging from 1.03 to 6.56 mmHg. Sobol analysis confirmed the dominant contribution of SR over PVR (S_1_: 0.856 vs. 0.034). Balloon wedging also induced measurable systemic perturbations, particularly under high-resistance conditions. Overall, HVPG is highly sensitive to SR but relatively insensitive to PVR, indicating a systematic underestimation risk in presinusoidal portal hypertension. Moreover, the hemodynamic perturbation induced by balloon wedging should not be neglected in severe portal hypertension, suggesting that this effect should be incorporated into virtual HVPG models to improve their predictive accuracy.

## 1. Introduction

Chronic liver disease (CLD) causes more than 2 million deaths each year, accounting for approximately 4% of global all-cause mortality [1]. Cirrhosis represents the end-stage manifestation of CLD, and the global prevalence of compensated cirrhosis was estimated at approximately 112 million cases in 2017 [2]. In cirrhosis and related disorders, structural remodeling of the hepatic sinusoids and fibrosis lead to a marked increase in intrahepatic vascular resistance, thereby elevating portal pressure and giving rise to a series of life-threatening complications, including gastroesophageal variceal bleeding, ascites, and hepatorenal syndrome [3,4].

**Figure 1:**
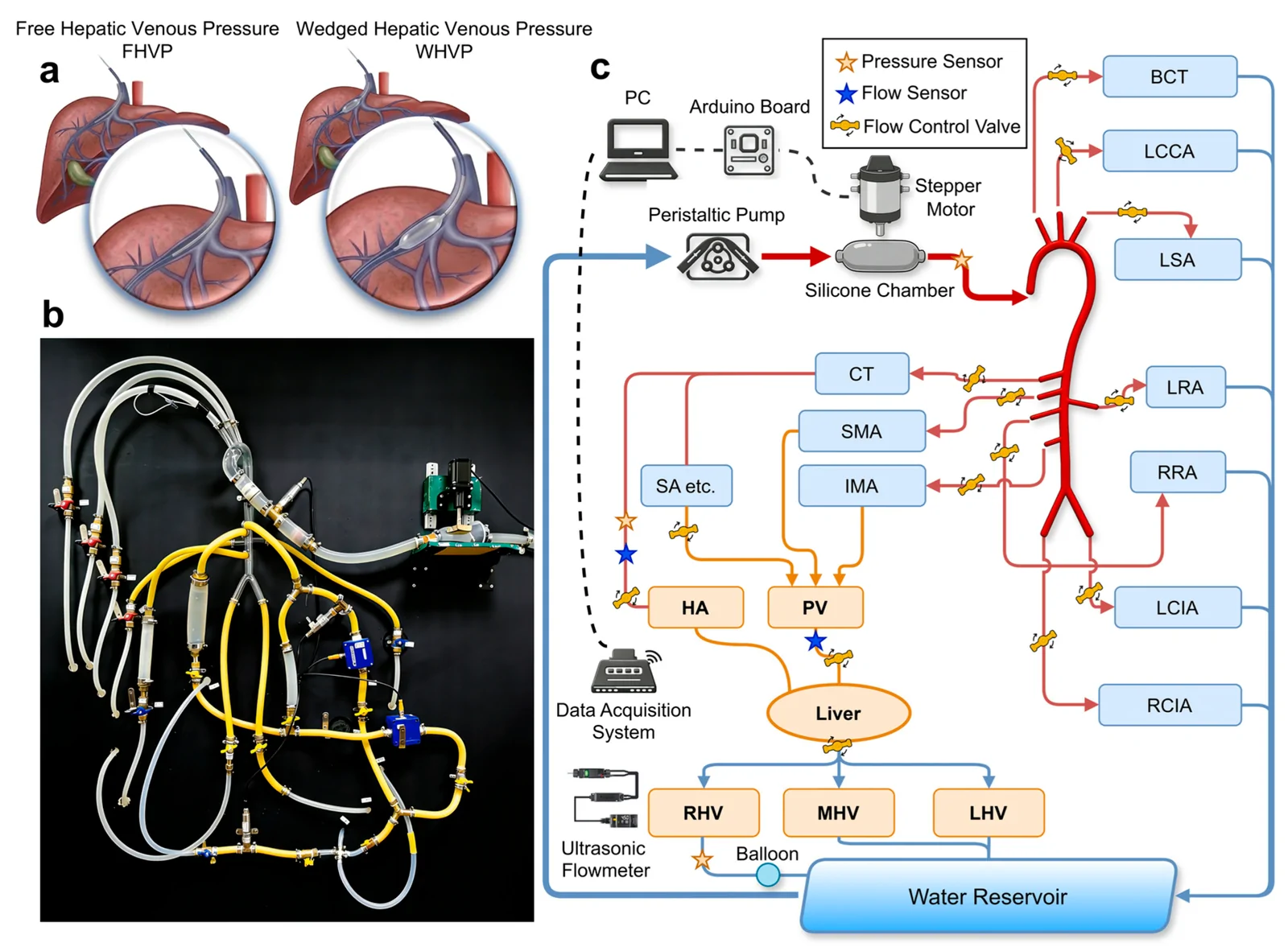
Schematic illustration of the clinical HVPG measurement principle and the experimental platform. (**a**) Clinical measurement of HVPG: a balloon catheter is advanced into the right hepatic vein via the jugular vein; wedged hepatic venous pressure (WHVP) is recorded under balloon inflation and occlusion, and free hepatic venous pressure (FHVP) is recorded after balloon deflation; HVPG = WHVP − FHVP. (**b**) Front-view photograph of the principal vascular and measurement components of the Mock Circulatory Loop (MCL). (**c**) Overall architecture of the in vitro MCL, including the Pulsatile Simulation Module, the Closed Circulatory Loop Module, and the Signal Acquisition Module. Vascular abbreviations: BCT, brachiocephalic trunk artery; LCCA, left common carotid artery; LSA, left subclavian artery; CT, celiac trunk; SMA, superior mesenteric artery; IMA, inferior mesenteric artery; LRA, left renal artery; RRA, right renal artery; SA, splenic artery; LCIA, left common iliac artery; RCIA, right common iliac artery; HA, hepatic artery; PV, portal vein; RHV, right hepatic vein; MHV, middle hepatic vein; LHV, left hepatic vein.

The hepatic venous pressure gradient (HVPG), defined as the difference between wedged hepatic venous pressure (WHVP) and free hepatic venous pressure (FHVP), is measured using a balloon catheter via the jugular vein or other venous access routes (Figure 1a). It is widely regarded as the gold standard for assessing portal hypertension and has important clinical value in disease staging and prognostic evaluation [5,6,7]. In healthy individuals, HVPG typically ranges from 2 to 5 mmHg; a value of ≥5 mmHg indicates portal hypertension, whereas a value of ≥10 mmHg defines clinically significant portal hypertension (CSPH) [6]. Although HVPG measurement is generally safe, it relies on invasive catheter-based techniques, which are costly, technically demanding, and associated with potential risks such as radiation exposure, hematoma, arrhythmia, and pneumothorax [5,8,9]. In addition, factors such as catheter position, respiratory variation, and systemic hemodynamic fluctuations may affect the stability and reproducibility of the measurements [10,11]. Porto-sinusoidal vascular disease (PSVD) causes predominantly presinusoidal portal hypertension, so hepatic venous pressure gradient (HVPG) often underestimates actual portal pressure [12]. Importantly, clinical studies have shown that HVPG exhibits differential sensitivity among portal hypertension subtypes; however, the underlying mechanisms remain unclear, and whether HVPG responds asymmetrically to different resistance components, such as portal venous resistance (PVR) and sinusoidal resistance (SR), has not been systematically investigated [13,14,15]. Baveno VII consensus recommends combining liver stiffness measurement and platelet count to rule out or rule in CSPH in patients with compensated advanced chronic liver disease [16]. This shift further emphasizes the need to define the physiological scope and limitations of HVPG as the invasive hemodynamic reference standard.

To overcome the limitations of invasive assessment, various noninvasive approaches have been developed in recent years, including transient elastography, imaging-based evaluation, and serum biomarkers [17,18,19]. Increased hydrodynamic pressure has been shown to impair the sinusoidal endothelium, with pressure-responsive molecular products proposed as potential biomarkers of portal hypertension [20]. However, these methods mainly rely on indirect indicators, making it difficult to accurately quantify portal pressure, and their accuracy remains controversial under complex pathological conditions [21]. Therefore, there is a clear need to develop an accurate in vitro approach for HVPG measurement. Among current strategies, virtual HVPG (vHVPG) based on CT angiography and computational fluid dynamics (CFD) has shown good agreement with invasive HVPG in multicenter validation studies, with an area under the curve (AUC) of up to 0.89 [22]. Nevertheless, the application of CFD models to the hepatic vasculature remains subject to several limitations, including insufficient segmentation accuracy for small branches, systematic errors introduced by rigid-wall assumptions, and limited generalizability due to small-sample validation [23]. In addition, clinical HVPG measurement depends on physical occlusion of the hepatic vein by a balloon catheter, whereas existing CFD methods usually simplify balloon wedging as a local boundary condition and implicitly assume that it has no significant effect on systemic hemodynamics [22,24,25]. More importantly, these approaches infer pressure indirectly from mathematical models and lack controlled experiments under physically constrained hemodynamic conditions.

In vitro experimental platforms provide another important means of investigating complex hemodynamic mechanisms. Compared with clinical studies and numerical simulations, in vitro systems offer both repeatability and physical realism, making them particularly suitable for studying a pressure-based hemodynamic index such as HVPG. Li et al. constructed an idealized in vitro portal vein model and used particle image velocimetry (PIV) to investigate flow characteristics in the portal venous system [26]. Rutkowski et al. developed a 3D-printed in vitro physical model of the portal vein based on the anatomy of living liver donors, integrated it into an extracorporeal perfusion loop, and used 4D Flow MRI together with flow probes to simultaneously measure flow fields and flow rates, thereby predicting hemodynamic changes associated with hepatectomy [27]. However, these in vitro platforms were based only on local portal venous models and did not incorporate the dual blood supply of the liver or the hepatic venous system, and thus could not enable in vitro measurement of HVPG.

To address these issues, the present study established an in vitro circulatory platform incorporating the dual hepatic blood supply. By modulating sinusoidal resistance and portal venous resistance, different degrees of portal hypertension were simulated, and HVPG was measured using a balloon wedging. The magnitude of systemic hemodynamic perturbation induced by balloon wedging was also analyzed. Furthermore, combined with global sensitivity analysis, the contributions of different resistance components to HVPG and related hemodynamic parameters were quantitatively evaluated, with particular emphasis on revealing the potential mechanism underlying its asymmetric sensitivity. This study aims to provide a hemodynamic basis for the clinical interpretation of HVPG and the optimization of measurement strategies, and to offer experimental support for the future development of noninvasive assessment techniques.

## 2. Methods

### 2.1. Experimental Platform Construction

In this study, we independently designed and constructed an in vitro Mock Circulatory Loop (MCL) with near-physiological hemodynamic characteristics to fully reproduce the human systemic circulation incorporating the liver’s dual blood supply (Figure 1b,c). The whole system consisted of three functional modules: a Pulsatile Simulation Module, a Closed Circulatory Loop Module, and a Signal Acquisition Module.

(1) Pulsatile Simulation Module: This module was jointly driven by a peristaltic pump (WT-600CAS/353Y-PPS, JIEHENG, Chongqing, China) and a stepper motor (23HS6401H-50D1020, GTW, Ningbo, China). An Arduino control board (UNO R3, ARDUINO, Monza, Italy) was programmed to precisely control the reciprocating motion of the stepper motor, which compressed a silicone chamber to simulate the periodic pumping process of the heart [28]. A one-way valve was installed at the aortic inlet to mimic the aortic valve and prevent backflow. The heart rate was set at 88 beats/min, and the ratio of systolic to diastolic duration was set to 24:44 (0.24 s:0.44 s). The baseline flow rate of the peristaltic pump was set to 5 L/min, corresponding to a stroke volume of approximately 57 mL, which is consistent with normal cardiac output under resting adult conditions [29].
(2) Closed Circulatory Loop Module: This module consisted of an aortic model and the connected vascular models. The aortic model was reconstructed in three dimensions based on the geometric parameters of an idealized adult aorta reported by Xiao et al. [30], and was fabricated from photosensitive resin using stereolithography apparatus (SLA). The model included the aorta major branches, including the brachiocephalic trunk artery, left common carotid artery, left subclavian artery, celiac trunk artery, superior mesenteric artery, inferior mesenteric artery, left and right renal arteries, and left and right common iliac arteries. In the hepatic vascular model, a three-way connector was used to divide the celiac trunk into a hepatic artery branch supplying arterial blood to the liver and other branches not directly entering the liver, such as the splenic artery and left gastric artery. Meanwhile, a portal vein model was constructed using the superior mesenteric vein and splenic vein as the main inflow branches, thereby fully reproducing the physiological structure of the liver’s dual blood supply. After the two inflow pathways merged, a valve was introduced to control sinusoidal resistance. Purified water was used as the working fluid, which is a commonly used substitute in current in vitro circulatory simulation studies because it is easy to prepare and clean and supports stable long-term operation [31,32,33]. Previous studies suggest that rheological assumptions have less influence on pressure-based indices than on local WSS [34]. Since this study focused on HVPG and its relative response to resistance changes rather than WSS, the use of water is unlikely to alter the main qualitative conclusions.
(3) Signal Acquisition Module: This module consisted of a high-precision pressure sensor (SCYG314, SEN, Wuxi, China), an electromagnetic flowmeter (FMG71B, OMEGA, Norwalk, CT, USA), a portable ultrasonic flowmeter (FDXA1/FDXS20/FDXC20R4, KEYENCE, Osaka, Japan), and a multifunction data acquisition system (USB3202, ART, Beijing, China). Signals were transmitted to a computer in real time via a USB interface and were synchronously acquired and displayed using LabVIEW (National Instruments, Austin, TX, USA). Pressure sensors were placed at three key measurement sites: the aortic inlet, the hepatic artery, and the right hepatic vein. Electromagnetic flowmeters were fixed at the hepatic artery and portal vein, respectively. The portable ultrasonic flowmeter could be flexibly positioned at the outlet of each branch. A balloon was placed in the right hepatic vein segment, and the clinical HVPG measurement procedure was simulated by balloon inflation and deflation to obtain wedged and free pressure measurements.

### 2.2. Calibration with Clinical Data

To ensure that the in vitro platform matched physiological conditions observed clinically, the MCL was systematically calibrated to near-physiological conditions before formal experiments. Based on the flow distribution data reported for the major arterial branches in healthy adults [30], the flow rate in each branch was finely adjusted using the portable ultrasonic flowmeter so that the measured flow distribution ratios matched the target values (Table 1). At the same time, physiological hepatic perfusion under the healthy baseline condition was ensured, with approximately 25% of total hepatic inflow supplied by the hepatic artery and 75% by the portal vein [35].

The systolic and diastolic pressures in the ascending aorta were required to remain within the normal physiological range (systolic pressure: 90–130 mmHg; diastolic pressure: 60–85 mmHg) [36,37]. The calibrated parameters were then used as the hemodynamic baseline for the subsequent resistance modulation experiments, providing a unified reference for quantifying the effects of resistance changes on HVPG.

### 2.3. Resistance Modulation Settings

This experiment focused on two core variables: SR, controlled by adjusting the valve downstream of the confluence of the hepatic artery and portal vein, and PVR, controlled by adjusting the valve in the portal venous pathway. To achieve quantifiable gradient control, a Relative Hepatic Perfusion Resistance Coefficient (*ζ*) was introduced and defined as follows:

(1) $$\zeta =\frac{R-R_{\min}}{R_{\max}-R_{\min}}$$

where *R* is the vascular resistance under the current condition, *R*_min_ is the resistance under the healthy baseline condition, and *R*_max_ is the resistance under severe cirrhosis or severe portal vein obstruction. Based on the range of hepatic vascular resistance reported in patients [13,38,39], the rotation angle of the valve was calibrated against the resistance coefficient, and nine gradient levels, *ζ*_1_*–ζ*_9_, were defined to represent different resistance conditions. Here, *ζ*_1_ corresponds to the healthy baseline state, whereas *ζ*_9_ corresponds to simulated portal hypertension under severe cirrhosis or severe portal vein obstruction.

By independently adjusting the resistances, portal hypertension states at different stages of liver disease progression were simulated. Under each resistance condition, the corresponding systemic hemodynamic variables were recorded and the associated HVPG values were calculated to construct the experimental dataset used for subsequent surrogate model fitting. The same physical MCL was used for all experimental conditions, without component replacement or reassembly between tests. The data were collected after the system had reached a stable operating state. This procedure was used to minimize potential inter-session mechanical drift and valve-setting hysteresis.

### 2.4. Data Signal Filtering

**Figure 2:**
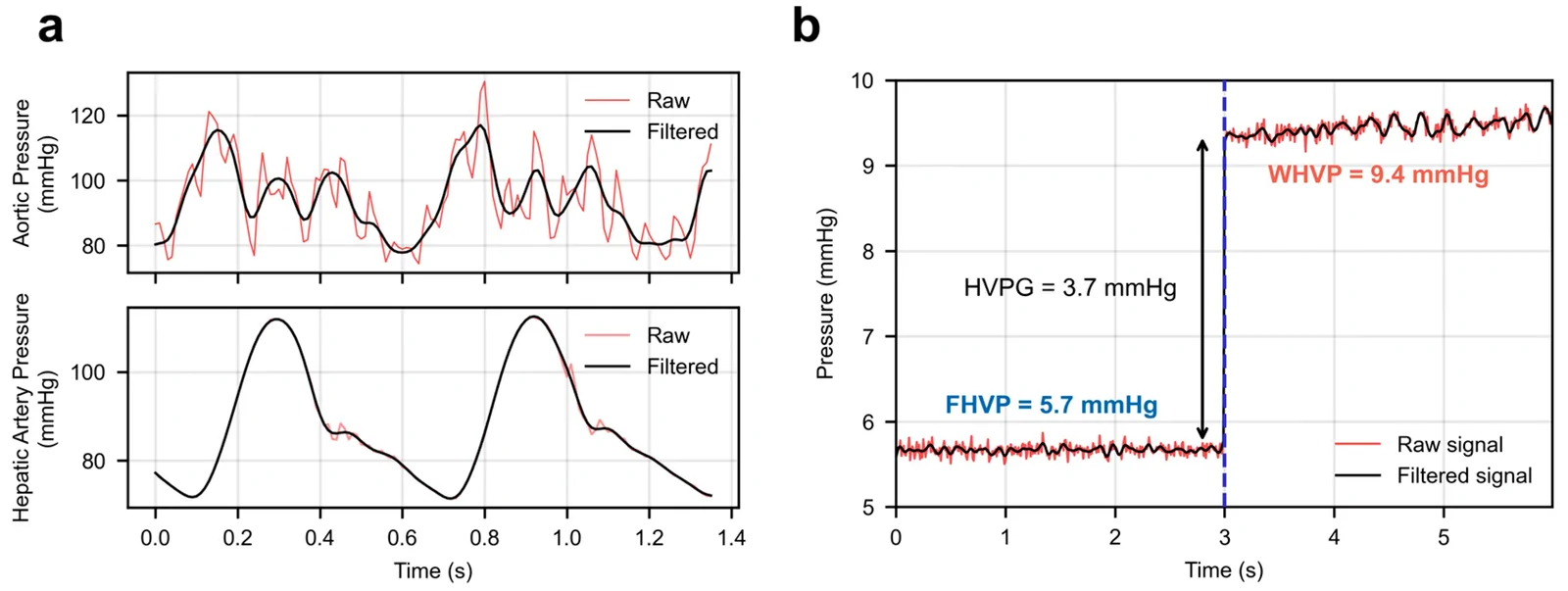
(**a**) Healthy baseline pressure waveform (Butterworth low-pass filter); (**b**) HVPG measurement under the healthy baseline condition.

The raw pressure and flow signals were acquired from the in vitro mechanical loop and inevitably contained noise components such as tubing vibration, electromagnetic interference, and nonphysiological high-frequency oscillations. To remove noise while preserving waveform morphology and peak locations, all pressure and flow signals were smoothed using a fourth-order zero-phase Butterworth low-pass filter, as shown in Figure 2.

The Butterworth filter is characterized by a flat frequency response, and the squared magnitude response of its transfer function is defined as [40]

(2) $$|H\left(j\omega \right){|}^{2}=\frac{1}{1+{\left(\frac{\omega }{\omega_{c}}\right)}^{2n}}$$

where *ω* is the angular frequency, *ω_c_* is the cutoff angular frequency, and *n* is the filter order, which was set to 4 in this study. A fourth-order Butterworth filter was selected to suppress high-frequency noise while preserving the principal physiological characteristics of the pressure and flow waveforms. For this reason, it is widely used in biomedical signal processing of cardiovascular pressure and flow signals [41].

The cutoff frequencies were selected according to physiological spectral characteristics: 12 Hz for pressure signals, 10 Hz for flow signals, and a system sampling frequency of 100 Hz. The major harmonic components associated with a heart rate of 88 beats/min (fundamental frequency, 1.47 Hz) were concentrated below 10 Hz, and a cutoff frequency of 12 Hz was sufficient to preserve systolic and diastolic pressure features. After filtering, one complete cardiac cycle was extracted for each experimental condition. The cycle-averaged value was used as the representative value, and the standard deviation of all sampling points within that cycle was used to construct the error bars, which characterize intra-cycle temporal variability associated with pulsatile flow.

### 2.5. Balloon Wedging Perturbation

#### 2.5.1. Hemodynamic State Vector

For each resistance condition, a hemodynamic state vector $V\in {\mathbb{R}}^{6}$ was defined for the system, whose components included the aortic pressure descriptors, hepatic arterial pressure, and the flow rates in the two hepatic inflow pathways monitored in the experimental platform:

(3) $$V={\left[P_{ao,sys},\;P_{ao,dia},\;P_{ao,mean},\;P_{ha},\;Q_{ha},\;Q_{pv}\right]}^{T}$$

where $P_{ao,sys}$, $P_{ao,dia}$ and $P_{ao,mean}$ represent the systolic, diastolic, and mean aortic pressures, respectively; $P_{ha}$ represents the mean hepatic arterial pressure; $Q_{ha}$ represents the mean hepatic arterial flow rate; and $Q_{pv}$ represents the mean portal venous flow rate.

The system state vectors under the balloon deflated state (free) and the balloon inflated occlusion state (wedge) are denoted as

(4) $$V_{free}={\left[P_{ao,sys}^{free},\;P_{ao,dia}^{free},\;P_{ao,mean}^{free},\;P_{ha}^{free},\;Q_{ha}^{free},\;Q_{pv}^{free}\right]}^{T}$$

(5) $$V_{wedge}={\left[P_{ao,sys}^{wedge},\;P_{ao,dia}^{wedge},\;P_{ao,mean}^{wedge},\;P_{ha}^{wedge},\;Q_{ha}^{wedge},\;Q_{pv}^{wedge}\right]}^{T}$$

The absolute perturbation vector ΔV induced by balloon wedging is defined as

(6) $$\Delta V=V_{wedge}-V_{free}$$

Because the components of the state vector have different physical dimensions and orders of magnitude (pressure in mmHg and flow in mL/min), direct comparison of their magnitudes would introduce dimensional bias and would not allow cross-comparison of the relative perturbations in pressure and flow variables. Therefore, the absolute perturbation vector was normalized using the corresponding components under the free state $V_{free}$ as the baseline, yielding the relative perturbation vector $\Delta V^{*}$:

(7) $$\Delta V^{*}=\Delta V⊘V_{free}={\left[\frac{\Delta P_{ao,sys}}{P_{ao,sys}^{free}},\;\frac{\Delta P_{ao,dia}}{P_{ao,dia}^{free}},\;\frac{\Delta P_{ao,mean}}{P_{ao,mean}^{free}},\;\frac{\Delta P_{ha}}{P_{ha}^{free}},\;\frac{\Delta Q_{ha}}{Q_{ha}^{free}},\;\frac{\Delta Q_{pv}}{Q_{pv}^{free}}\right]}^{T}$$

Each component of the $\Delta V^{*}$ is a dimensionless relative change; a positive value indicates an increase in that parameter after balloon wedging, whereas a negative value indicates a decrease.

#### 2.5.2. Perturbation Index

To compress the six-dimensional relative perturbation vector into a single quantifiable scalar that collectively characterizes the magnitude of systemic perturbation induced by balloon wedging, the Perturbation Index (PI) was defined as the Euclidean norm of the relative perturbation vector:

(8) $$\mathrm{PI}=∥\Delta V^{*}{∥}_{2}=\sqrt{\sum_{i}{\left(\Delta V_{i}^{*}\right)}^{2}}$$

PI represents the displacement distance of the system state caused by balloon wedging in a six-dimensional normalized parameter space. PI = 0 corresponds to the implicit assumption adopted by current virtual HVPG methods, namely that balloon wedging causes no perturbation at all to systemic circulatory variables. A larger PI indicates a stronger overall hemodynamic disturbance induced by balloon wedging.

### 2.6. Sensitivity Analysis Methods

#### 2.6.1. Radial Basis Function Surrogate Model

Because the number of experimental sampling points was limited, direct global sensitivity analysis based on discrete data points would have restricted statistical power. Therefore, a Radial Basis Function (RBF) was used to interpolate the experimental data and construct a continuous surrogate model of HVPG as a function of the two resistance parameters [42]. The general form of the RBF surrogate model is

(9) $$\hat{y}\left(x\right)=\overset{N}{\underset{i=1}{\sum }}w_{i}\;\varphi \;\left(∥x-x_{i}∥\right)$$

where $x={\left(\zeta_{\mathrm{SR}},\;\zeta_{\mathrm{PVR}}\right)}^{T}$ denotes the two-dimensional input variable, $x_{i}$ denotes the i-th experimental sample point, and a thin-plate spline kernel ($\varphi \left(r\right)=r^{2}\ln r,\;r=\|x-x_{i}{\|}_{2}$) was adopted as the basis function in this study. The weight coefficients $w_{i}$ were determined by solving the interpolation equations based on the experimental samples, and *N* is the total number of samples. The RBF model provides interpolation accuracy and generalization ability for sparse, unstructured sampling points.

#### 2.6.2. Sobol Global Sensitivity Analysis

After generating a dense set of sampled points from the RBF surrogate model, Sobol’ Global Sensitivity Analysis was used to quantify the contributions of SR and PVR to each output variable [43]. The predictive fidelity of each RBF surrogate model was evaluated using leave-one-out cross-validation (LOOCV). In each of 17 iterations, one of the 17 unique experimental input combinations was withheld, the RBF surrogate model was refitted using the remaining 16 combinations, and the response at the withheld combination was predicted. The root-mean-square error (RMSE) for each output was calculated from the 17 pooled out-of-sample predictions. The Sobol method is based on a variance-decomposition framework, in which the total output variance $Var\left(Y\right)$ is decomposed into contributions from the input variables:

(10) $$Var\left(Y\right)=\underset{i}{\sum }V_{i}+\underset{i<j}{\sum }V_{ij}+\ldots +V_{12\ldots k}$$

where $V_{i}=Var\;\left[E\left(Y|X_{i}\right)\right]$ denotes the first-order contribution variance of the i-th variable and $V_{ij}$ denotes the interaction term. Accordingly, the first-order Sobol index is defined as

(11) $$S_{i}=\frac{V_{i}}{Var\left(Y\right)}=\frac{Var\;\left[E\left(Y|X_{i}\right)\right]}{Var\left(Y\right)}$$

and the total-order Sobol index is defined as

(12) $$S_{T_{i}}=1-\frac{Var\;\left[E\left(Y|X_{∼i}\right)\right]}{Var\left(Y\right)}$$

where $X_{∼i}$ denotes the set of all input variables except $X_{i}$, with $S_{T_{i}}$ capturing both the main effect and higher-order interaction effects.

Using HVPG, WHVP, FHVP, hepatic arterial pressure, and aortic pressure as output variables, and the sinusoidal resistance coefficient (*ζ_SR_*) and portal venous resistance coefficient (*ζ_PVR_*) as input variables, the corresponding Sobol indices ($S,\;S_{T}$) were calculated for each output to quantitatively compare the asymmetric sensitivity of HVPG to variations in the two resistance components. The uncertainty of the Sobol indices was estimated using 1000 bootstrap resamples at a confidence level of 95%, conditional on the fitted RBF surrogate model.

## 3. Results

### 3.1. Baseline Hemodynamic Characteristics

Under the baseline condition (*ζ*_1_), the ascending aortic pressure waveform in the in vitro Mock Circulatory Loop exhibited typical physiological pulsatile characteristics, with a systolic pressure of 117 mmHg and a diastolic pressure of 78 mmHg, both within the normal adult range (systolic pressure: 90–130 mmHg; diastolic pressure: 60–85 mmHg) [36,37]. The central venous return pressure was approximately 5 mmHg [44] (Figure 2a). The flow distribution ratios of the major arterial branches were generally consistent with the reference values reported in the literature [30] (Table 1). In particular, the flow ratio between the hepatic artery and portal vein was approximately 1:3.2, which is consistent with the physiological characteristics of the liver’s dual blood supply [35,45]. It should be noted that, as in many currently reported in vitro studies, the aortic pressure waveform obtained in the present study contained some non-physiological oscillations [31,46,47,48]. These discrepancies may be attributed to the purely elastic behavior of the 3D-printed aortic wall [47]. In addition, vibration of the stepper motor may also produce multiple high-frequency oscillations within each cardiac cycle [31]. Nevertheless, compared with the sinusoidal blood pressure waveform generated by a blood pump [49], the waveform obtained here was closer to the true physiological waveform. Since the present study mainly focused on HVPG measurement and such oscillations were difficult to propagate to the hepatic vein, they were not expected to affect the final experimental results.

### 3.2. Systemic Hemodynamic Responses

**Figure 3:**
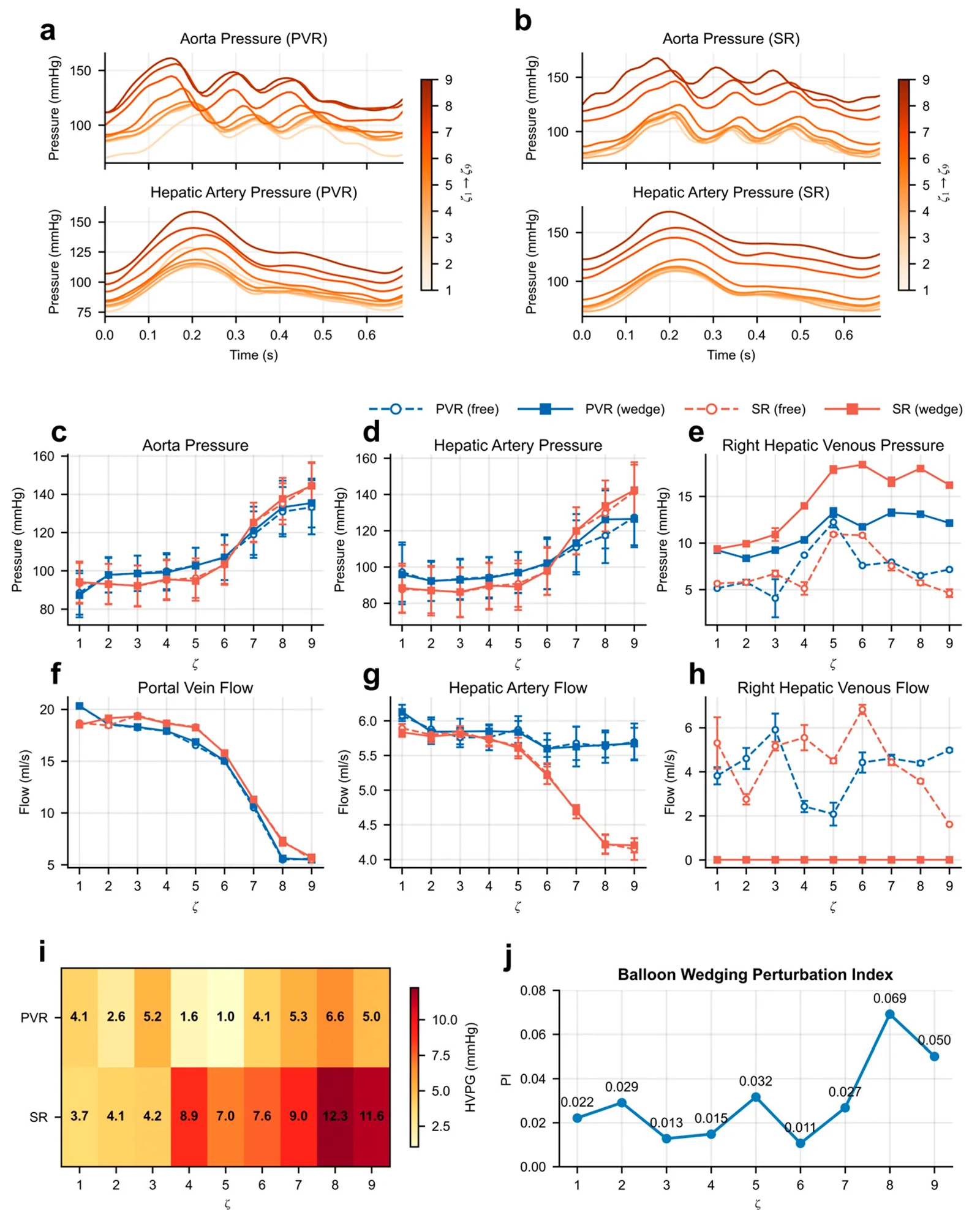
(**a**) Comparison of aortic and hepatic arterial pressure waveforms under different PVR coefficients; (**b**) comparison of aortic and hepatic arterial pressure waveforms under different SR coefficients; (**c**–**e**) changes in mean aortic pressure, mean hepatic arterial pressure, and mean right hepatic venous pressure as a function of *ζ* (PVR group: blue; SR group: orange; free state: dashed lines with circles; wedge state: solid lines with squares), where the error bars indicate the standard deviation within one cardiac cycle; (**f**–**h**) changes in mean portal venous flow, mean hepatic arterial flow, and mean right hepatic venous flow as a function of *ζ,* where the error bars indicate the standard deviation within one cardiac cycle; (**i**) heatmap of HVPG values in the SR and PVR groups; (**j**) variation in the perturbation index PI with *ζ*.

Figure 3a–h summarizes the changes in systemic hemodynamic parameters under two independently controlled resistance conditions SR and PVR, across *ζ*_1_–*ζ*_9_. These parameters included the pressure waveforms and mean pressures of the aorta and hepatic artery, the mean flow rates of the portal vein and hepatic artery, and the changes in free hepatic venous pressure and wedged hepatic venous pressure before and after balloon wedging.

Under both resistance conditions, the pulsatile pressure waveforms showed a trend toward increased systolic peak pressure and widened pulse pressure with increasing resistance coefficient (Figure 3a,b). As SR increased, the mean aortic pressure rose monotonically from 93.4 mmHg at baseline *ζ*_1_ to 144.8 mmHg at *ζ*_9_, corresponding to a total increase of approximately 55.0%. Meanwhile, the mean hepatic arterial pressure increased from 87.6 mmHg to 141.6 mmHg, corresponding to an increase of approximately 61.6%. In contrast, as PVR increased, the mean aortic pressure rose from 88.6 mmHg to 133.2 mmHg, corresponding to an increase of approximately 50.3%, while the mean hepatic arterial pressure increased from 97.2 mmHg to 127.4 mmHg, corresponding to an increase of approximately 31.1%. Compared with the SR group, the PVR group showed a similar overall upward trend in aortic and hepatic arterial pressures, but with a smaller magnitude of increase (Figure 3c,d).

As SR increased, the mean hepatic arterial flow decreased from 5.89 mL/s to 4.15 mL/s, corresponding to a reduction of approximately 29.5%, whereas the mean portal venous flow decreased from 18.69 mL/s to 5.50 mL/s, corresponding to a reduction of approximately 70.6%. These results indicate a highly coordinated downward trend, with the reduction in portal venous flow being much greater than that in hepatic arterial flow. This suggests that, when sinusoidal resistance is the dominant factor, the reduction in hepatic inflow occurs primarily on the portal venous side. As PVR increased, hepatic arterial flow remained relatively stable, decreasing only from 6.08 mL/s to 5.70 mL/s, corresponding to a reduction of approximately 6.3%, whereas portal venous flow decreased from 20.33 mL/s to 5.59 mL/s, corresponding to a reduction of approximately 72.5%, which was comparable to the decrease observed in the SR group. Notably, hepatic arterial flow in the PVR group was scarcely affected, indicating a relative independence of hepatic arterial flow from changes in PVR (Figure 3f,g).

### 3.3. HVPG Measurement

As shown in Figure 3e, under the free state, the pressure in the right hepatic vein remained at a relatively low level overall (approximately 5–11 mmHg), with only a local increase around the intermediate resistance level *ζ*_5_, followed by a slight decline. This suggests that, under the free state, hepatic venous pressure responded only modestly to changes in resistance. In contrast, under the wedge state, the pressure increased with increasing *ζ*. Although slight fluctuations were observed after *ζ*_5_, it remained at a relatively high level overall. Further comparison between the two resistance mechanisms showed that the increase in pressure in the SR group was substantially greater than that in the PVR group, and that the SR group exhibited a more pronounced pressure accumulation effect at high resistance levels.

As shown in Figure 3h, under the wedge state, flow in the right hepatic vein was nearly zero at all resistance levels and did not vary with *ζ*, indicating that this state effectively blocked blood flow and allowed the measurement to primarily reflect static pressure characteristics, thereby supporting the validity of the experimental setup.

The heatmap in Figure 3i presents the distribution of HVPG values under the two resistance types across the nine resistance levels. A marked contrast was observed between the two groups. In the SR modulation group, HVPG showed a clear monotonic increase with increasing sinusoidal resistance, rising from 3.69 mmHg at *ζ*_1_ to a maximum of 12.25 mmHg at *ζ*_8_, followed by a slight decrease to 11.58 mmHg at *ζ*_9_. HVPG first exceeded 5 mmHg at *ζ*_4_ (8.86 mmHg), indicating the onset of portal hypertension. At *ζ*_8_, it exceeded the threshold for clinically significant portal hypertension (CSPH, ≥10 mmHg [16]) and also crossed the threshold associated with variceal bleeding risk (≥12 mmHg [16]), reaching 12.25 mmHg.

In the PVR modulation group, HVPG did not exhibit a monotonic trend and fluctuated within the range of 1.03–6.56 mmHg. None of the nine resistance levels exceeded the threshold of 10 mmHg for CSPH.

### 3.4. Balloon Wedging PI

Figure 3j shows the variation in the PI with the resistance coefficient. PI was greater than zero under all experimental conditions (range: 0.011–0.069), indicating that balloon wedging was not entirely without effect on systemic circulatory parameters.

Overall, PI increased with increasing *ζ* and reached its peak at *ζ*_8_ (PI = 0.069), followed by a value of 0.050 at *ζ*_9_. In the low-resistance range (*ζ*_1_–*ζ*_6_), PI remained at a relatively low level (0.011–0.032), whereas a marked increase was observed in the high-resistance range (*ζ*_7_–*ζ*_9_). These results suggest that, at more severe levels of portal hypertension, the perturbation induced by balloon wedging on the systemic circulation becomes substantially more pronounced.

### 3.5. Global Sensitivity Analysis

#### 3.5.1. HVPG Response Surface

**Figure 4:**
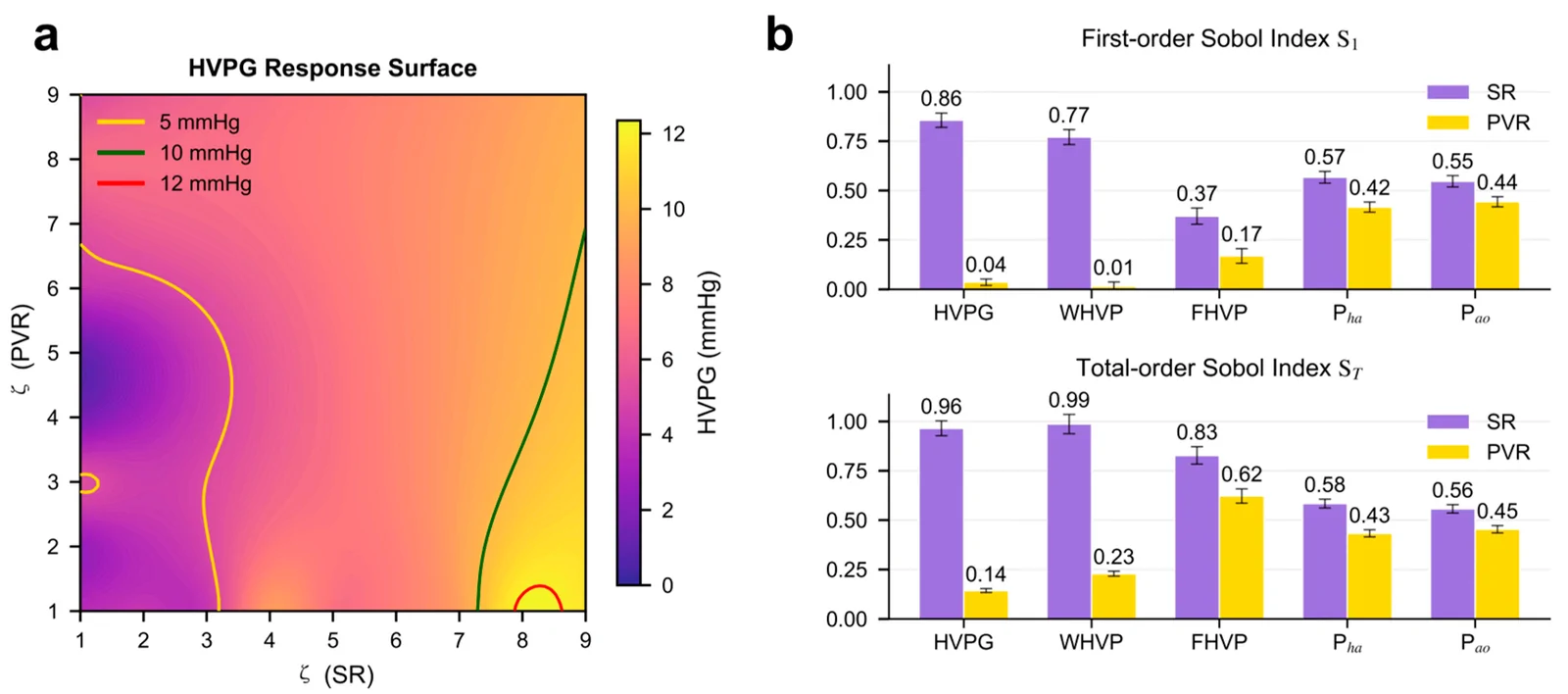
Sensitivity analysis results: (**a**) HVPG response surface based on the RBF surrogate model. The x-axis represents *ζ* (SR), and the y-axis represents *ζ* (PVR). Three clinical threshold contours are labeled at 5 mmHg (onset of portal hypertension), 10 mmHg (clinically significant portal hypertension, CSPH), and 12 mmHg (risk threshold for variceal bleeding). (**b**) Bar plots of Sobol global sensitivity analysis results. The upper panel shows the first-order sensitivity index (*S*_1_), and the lower panel shows the total-order sensitivity index (*S*_T_). The x-axis represents five output variables (HVPG, WHVP, FHVP, *P_ha_*, and *P_ao_*), and the y-axis represents Sobol index values (0–1). Purple bars indicate the contribution of SR, and yellow bars indicate the contribution of PVR.

The HVPG response surface generated from the RBF surrogate model (Figure 4a) provides an intuitive visualization of the distribution of HVPG in the two-dimensional SR–PVR parameter space. Overall, the response surface exhibited a steep gradient along the SR axis and a much weaker gradient along the PVR axis, further confirming, from the perspective of parameter space, the dominant sensitivity of HVPG to SR and its insensitivity to PVR. The three clinical threshold contours (5, 10, and 12 mmHg) were distributed mainly along the SR axis. In the lower-left region of the response surface, HVPG remained largely below 5 mmHg, corresponding to the threshold for the onset of portal hypertension. In the lower-right region, the response surface crossed the 10 mmHg threshold for CSPH, and a small portion further entered the region above 12 mmHg, corresponding to the risk threshold for variceal bleeding. Most of the response surface lay within the range of 5–10 mmHg. LOOCV yielded RMSE values of 2.071, 1.455, 1.994, 2.883, and 3.582 mmHg for HVPG, WHVP, FHVP, *P*_ha_, and *P*_ao_, respectively. These results provide a quantitative assessment of the predictive fidelity of the RBF surrogate models.

#### 3.5.2. Sobol Global Sensitivity Indices

Table 2 presents the first-order Sobol indices (*S*_1_) and total-order Sobol indices S_T_ for five output variables: HVPG, WHVP, FHVP, *P*_ha_, and *P*_ao_. Figure 4b provides a graphical comparison of these indices. The error bars represent bootstrap-based 95% confidence intervals obtained using 1000 resamples. These intervals quantify the sampling uncertainty of the Sobol estimators conditional on the fitted RBF surrogate and do not include between-run experimental uncertainty because independent experimental replicates were unavailable.

For HVPG, the first-order sensitivity index of $S_{1}^{SR}$ = 0.856, whereas that $S_{1}^{PVR}$ = 0.034, yielding a ratio of approximately 25:1. This quantitatively demonstrates the high sensitivity of HVPG to SR and its marked insensitivity to PVR. The corresponding 95% confidence intervals were 0.816–0.896 for SR and 0.018–0.050 for PVR. The clear separation between these intervals indicates that the dominant contribution of SR was stable with respect to the sampling uncertainty of the Sobol estimator. WHVP showed a similar asymmetric pattern to HVPG ($S_{1}^{SR}$ = 0.774, $S_{1}^{PVR}$ = 0.014), whereas the sensitivity distribution of FHVP was relatively more balanced ($S_{1}^{SR}$ = 0.377, $S_{1}^{PVR}$ = 0.174), suggesting that the difference in response of FHVP to the two resistance components was smaller than that of WHVP.

Notably, the sensitivity distributions of *P_ha_* and *P_ao_* were highly balanced. For *P_ha_*, the first-order indices $S_{1}^{SR}$ = 0.567 and $S_{1}^{PVR}$ = 0.416; for *P_ao_*, the corresponding values $S_{1}^{SR}$ = 0.546 and $S_{1}^{PVR}$ = 0.443. The ratios were close to 1.25:1, indicating that systemic arterial pressure did not exhibit a clear preference for either resistance component. This suggests that the asymmetric sensitivity of HVPG is a local characteristic specific to the hepatic venous pressure measurement system, rather than a general feature of the overall systemic hemodynamic response.

The total-order Sobol index *S*_T_ further reflects the overall dependence of each output variable on the two input variables, including both main effects and higher-order interaction effects. For HVPG, the total-order index $S_{T}^{SR}$ = 0.964, much higher than that $S_{T}^{PVR}$ = 0.144, indicating that the dominant role of SR in determining HVPG remained highly significant even after interaction effects were taken into account. This result suggests that the response of HVPG to SR was robust and consistent throughout the parameter space and was not attenuated by changes in PVR. WHVP showed a similar pattern in its total-order indices ($S_{T}^{SR}$ = 0.988, $S_{T}^{PVR}$ = 0.229); $S_{T}^{SR}$ was close to 1 which indicates that SR alone accounted for nearly all of the variance in WHVP. For FHVP, both total-order indices were relatively high ($S_{T}^{SR}$ = 0.826, $S_{T}^{PVR}$ = 0.625), and their sum (1.451) substantially exceeded 1, indicating a strong nonlinear interaction between the two resistance components in determining FHVP. For *P_ha_* and *P_ao_*, the total-order indices were approximately 0.584/0.433 and 0.557/0.454, respectively. In both cases, the sums of the *S*_T_ values were close to 1 (approximately 1.01), indicating minimal interaction effects and suggesting that the responses of systemic arterial pressure to SR and PVR were essentially linearly additive, without significant synergistic or antagonistic interactions.

The difference between *S*_T_ and *S*_1_ reflects the interaction effect between the two input variables. The interaction term was approximately 0.107 for HVPG and 0.214 for WHVP, both relatively small, indicating that the effects of SR and PVR on HVPG and WHVP were dominated by independent main effects, with interaction effects playing a secondary role. In contrast, the interaction term for FHVP was relatively large (approximately 0.45), suggesting that FHVP was more strongly influenced by nonlinear coupling between the two resistance components. For *P_ha_* and *P_ao_*, the interaction terms were both below 0.03 and were therefore almost negligible.

## 4. Discussion

The in vitro MCL developed in this study achieved the central objective of measuring HVPG under controlled physical conditions. The baseline calibration results were generally consistent with clinical data: the aortic systolic pressure of 117 mmHg and diastolic pressure of 78 mmHg both fell within the normal physiological range, and the deviations in flow distribution ratios among the major arterial branches remained within reasonable limits, thereby supporting the near-physiological validity of the in vitro platform [36,37].

The three core findings of this study can be summarized as follows. (1) SR is the decisive factor driving the increase in HVPG: the first-order Sobol index $S_{1}^{SR}$ = 0.856, and its contribution to the variance of HVPG was approximately 25 times that of PVR ($S_{1}^{PVR}$ = 0.034). This quantitative difference provides the most direct in vitro experimental evidence to date for the asymmetric sensitivity of HVPG. (2) PVR had little effect on HVPG, but its influence on systemic arterial pressure (*P_ha_* and *P_ao_*) was comparable to that of SR (sensitivity ratio approximately 1:1.25), indicating a systematic “blind spot” of HVPG measurement with respect to changes in resistance on the portal venous side. (3) Balloon wedging did not cause zero perturbation to the systemic circulation (maximum PI = 0.069), and the magnitude of the perturbation increased with the severity of portal hypertension. This effect is ignored by current virtual HVPG models and may therefore represent a potential source of systematic error.

### 4.1. Effects of Resistance Modulation on Systemic Hemodynamics

In the present study, increases in both SR and PVR led to systematic elevations in aortic and hepatic arterial pressures. As hepatic vascular resistance increased, hepatic inflow decreased, whereas cardiac output remained constant. This elevated peripheral arterial pressure. In terms of local flow, portal venous flow decreased markedly with increasing resistance in both conditions, with a reduction of approximately 70%. In contrast, hepatic arterial flow showed only a mild decrease under increased PVR (approximately 6.3%) and remained relatively stable overall. It should be noted that this behavior did not reproduce the classical hepatic arterial buffer response (HABR), which is characterized by a decrease in portal venous flow accompanied by compensatory increases in hepatic arterial flow [50,51]. Instead, the results suggest that, within the current in vitro platform, purely mechanical resistance modulation can induce only limited flow redistribution and is insufficient to trigger a substantial compensatory increase in hepatic arterial flow. Therefore, this phenomenon is better interpreted as a limited passive stabilization of hepatic blood supply rather than a direct reproduction of classical HABR. The active adenosine-mediated regulatory component of HABR may still be essential for achieving a true increase in hepatic arterial flow.

### 4.2. Mechanistic Interpretation and Clinical Significance of the Asymmetric Sensitivity of HVPG

The most important finding of this study is the asymmetric sensitivity of HVPG to SR and PVR ($S_{1}^{SR}$/$S_{1}^{PVR}$ ≈ 25). From the measurement principle, HVPG = WHVP − FHVP, where WHVP reflects sinusoidal pressure after balloon occlusion of the hepatic vein, whereas FHVP reflects pressure on the inferior vena cava side. SR acts directly on the pressure node sensed by WHVP. Therefore, when SR increases, WHVP rises markedly while FHVP changes only slightly, resulting in a substantial increase in HVPG [52,53].

In contrast, PVR is located upstream of the hepatic sinusoids. When PVR increases, portal venous pressure also rises. However, as this pressure signal is transmitted through the sinusoids toward WHVP, it is buffered by the sinusoidal compartment and constrained by the pressure balance with the hepatic artery, so that WHVP does not increase proportionally. At the same time, FHVP also changes in response to systemic pressure fluctuations, causing the HVPG difference to remain relatively stable or even decrease. This mechanism is fully consistent with the clinical observation that HVPG is relatively insensitive to presinusoidal portal hypertension, such as portal vein thrombosis or schistosomiasis, but highly sensitive to sinusoidal portal hypertension, such as cirrhosis [54,55,56]. Therefore, for diseases in which increased PVR is the dominant pathological feature, HVPG may underestimate the actual portal pressure. Clinicians should therefore interpret HVPG values in such patients with caution and combine them with other radiological or pathological indicators to avoid missing the diagnosis of portal hypertension on the basis of a “normal” HVPG alone [57,58]. HVPG should be interpreted together with liver stiffness, platelet count, imaging findings, and, when clinically indicated, alternative or direct assessments of portal pressure rather than being considered in isolation. Importantly, the Baveno VII thresholds were developed primarily for compensated advanced chronic liver disease and should not be directly extrapolated to predominantly presinusoidal disorders such as portal vein thrombosis, schistosomiasis, or porto-sinusoidal vascular disease [16].

The high sensitivity of HVPG to SR also provides mechanistic support for therapies aimed at reducing intrahepatic resistance, particularly carvedilol [59,60], and possibly adjunctive agents such as statins [61], which may improve intrahepatic vascular resistance and thereby contribute to portal pressure reduction.

Sobol analysis showed that FHVP responded relatively evenly to the two resistance components ($S_{1}^{SR}$ = 0.377, $S_{1}^{PVR}$ = 0.174), whereas FHVP is often treated in clinical practice merely as a reference zero point and has not received sufficient attention. Previous studies have suggested that FHVP measurement depends on catheter tip position and venous morphology and may show poor consistency [62]. Therefore, independent changes in FHVP deserve closer attention in HVPG analysis.

It is worth comparing these findings with previous computational models. Wang et al. [63,64] numerically investigated HVPG sensitivity using a lumped-parameter model and concluded that sinusoidal resistance exerts a stronger influence than presinusoidal resistance, which is directionally consistent with the in vitro results of the present study. However, their numerical model could not reproduce the three-dimensional physical process of balloon wedging. The present experimental platform provides an independent experimental validation of these mechanisms under real physical constraints and thus offers a benchmark for calibrating future computational models.

### 4.3. Perturbation Induced by Balloon Wedging and Its Implications for Virtual HVPG Methods

This study is the first to quantitatively characterize, at the level of in vitro hemodynamics, the perturbation induced by balloon wedging on the systemic circulation (PI: 0.011–0.069). Although the absolute values of PI appear limited, an important point should be emphasized: the perturbation increased with the severity of portal hypertension, with a marked rise in the high-resistance range (*ζ*_7_–*ζ*_9_). This implies that in patients with severe portal hypertension, for whom accurate HVPG measurement is most clinically important, the systemic perturbation caused by balloon wedging may in fact be the greatest. This finding has direct methodological implications for current studies on virtual HVPG. For example, Qi et al. [22] developed a computational virtual HVPG approach based on three-dimensional reconstruction and CFD. Qiu et al. [24,25] modeled the liver region as porous media and used CFD to noninvasively estimate the HVPG and portal pressure gradient. In these CFD models, wedged-pressure boundary conditions are imposed as hard boundary conditions, implicitly assuming that balloon occlusion does not alter the upstream hemodynamic environment. The present findings indicate that treating balloon wedging as hemodynamically neutral may introduce non-negligible error under high-resistance conditions (ζ_7_–ζ_9_). Future virtual HVPG models intended to reproduce invasive balloon-occlusion measurements should therefore evaluate balloon–flow coupling effects, particularly in simulations of clinically significant portal hypertension (HVPG ≥ 10 mmHg). Once validated against independent experimental and clinical data, a perturbation correction may improve agreement between virtual and invasive HVPG measurements.

### 4.4. Limitations and Future Work

Several limitations of this study should be acknowledged. First, the aortic model in this platform was fabricated using stereolithography resin, whose compliance is lower than the physiological level of real vessels and may therefore influence pressure-wave propagation and pulsatile waveform characteristics [65,66]. Second, purified water was used as the working fluid and thus did not reproduce the non-Newtonian rheological behavior of blood; this simplification may introduce bias under low-shear or microvascular conditions [67,68]. Nevertheless, these simplifications help isolate the dominant effects of resistance changes under controlled conditions. In addition, the present study adopted an independent sampling strategy for SR and PVR and therefore did not fully cover the parameter space in which both rise simultaneously. Future work will incorporate more physiologically realistic vascular compliance and fluid properties and will expand the sampling range to improve the physiological fidelity and reliability of the model.

The platform may also be extended in several directions. (1) By integrating an extracorporeal liver support system [69] and introducing PID-based active control, it may become possible to simulate the dynamic compensatory response of the hepatic artery and thereby achieve resistance modulation closer to clinical reality. (2) By combining the in vitro application of PIV [26,70] with comparison against clinical 4D-flow MRI, the platform may enable visual validation of three-dimensional flow fields and quantitative analysis of flow field characteristics [71]. (3) On the basis of the current platform, a “mechanical twin” system could be developed through high-fidelity parameter calibration to establish personalized in vitro models corresponding to specific patient pathophysiological states, such as compliance-specific aortic models and conduits, thereby exploring the possibility of preoperative hemodynamic prediction.

## 5. Conclusions

This study systematically elucidated the mechanism underlying the asymmetric sensitivity of HVPG in vitro hemodynamic environment. The results show that HVPG is determined primarily by sinusoidal resistance and is relatively insensitive to changes in portal venous resistance, thereby explaining its diagnostic limitation in presinusoidal portal hypertension and underscoring the need for integrated interpretation with other radiological or pathological indicators. In addition, balloon wedging induces a quantifiable perturbation in the systemic circulation, and this perturbation increases with the severity of portal hypertension, indicating that this effect should not be ignored in current virtual HVPG models. From the perspective of physical mechanism, this study provides an important basis for the clinical interpretation of HVPG and for the optimization of noninvasive assessment methods. Future integration of in vitro experiments with imaging data and numerical simulations may further promote the development of HVPG-related noninvasive assessment toward greater accuracy and clinical translation.

## Figures and Tables

**Table 1:** Diameter and calibrated flow distribution ratios of the major arterial branches.

Vessel Branch	Diameter (mm)	Measured Flow Distribution Ratio (%)	Target Flow Distribution Ratio (%)
Ascending aorta (inlet)	30.5	100.0	100
Brachiocephalic trunk artery	12.7	13.2	10.41
Left common carotid artery	7.2	2.5	2.14
Left subclavian artery	9.6	7.1	8.27
Celiac trunk artery	8.9	15.3	13.24
Superior mesenteric artery	7.5	12.1	15.97
Inferior mesenteric artery	4.0	2.0	2.16
Left renal artery	5.6	13.2	13.15
Right renal artery	5.6	12.8	13.15
Left common iliac artery	12.0	11.7	10.76
Right common iliac artery	12.0	10.1	10.76

**Table 2:** First-order and total-order Sobol sensitivity indices for the five hemodynamic outputs.

Output Variable	SR (S_1_)	PVR (S_1_)	SR (S_T_)	PVR (S_T_)
HVPG	0.856 ± 0.040	0.034 ± 0.016	0.964 ± 0.039	0.144 ± 0.010
WHVP	0.774 ± 0.040	0.014 ± 0.023	0.988 ± 0.050	0.229 ± 0.013
FHVP	0.377 ± 0.040	0.174 ± 0.038	0.826 ± 0.043	0.625 ± 0.040
*P*_ha_	0.567 ± 0.029	0.416 ± 0.026	0.584 ± 0.023	0.433 ± 0.019
*P*_ao_	0.546 ± 0.029	0.443 ± 0.026	0.557 ± 0.022	0.454 ± 0.019

Note: Values are presented as the Sobol index estimate ± the 95% confidence-interval half-width. S_1_ denotes the first-order Sobol index, and S_T_ denotes the total-order Sobol index. Confidence intervals were obtained using 1000 bootstrap resamples and quantify the sampling uncertainty of the Sobol estimators conditional on the fitted RBF surrogate.

## Data Availability

The data that support the findings of this study are available from the corresponding author upon reasonable request.
